# Distinctive transnational city-to-city partnerships, decentralization, and local governance of China as a Global East Country

**DOI:** 10.1371/journal.pone.0288001

**Published:** 2023-07-21

**Authors:** Jili Xu, Huaikuan Liu, Gengzhi Huang

**Affiliations:** 1 Guangzhou Institute of Geography, Guangdong Academy of Sciences, Guangzhou, China; 2 Dongguan Urban Planning and Design Institute, Dongguan, China; 3 School of Geography and Planning, Sun Yat-sen University, Guangzhou, China; East China Normal University, CHINA

## Abstract

Against the background of globalisation and state rescaling, promoting decentralisation and enhancing local governance capacity have become prioritised objectives of transnational city-to-city partnerships mainly between developed and developing countries. However, considering the critical debates on Global East’s uniqueness, two questions emerge when studying the transnational partnerships of Chinese cities. (1) Are Chinese cities’ partnership establishments and objectives remarkably different from the existing international body of knowledge? (2) In China, whether decentralisation and local governance are promoted by city-to-city transnational partnerships as well? To cope with the questions, this paper examines 28 Chinese world cities’ partnership establishments and objectives and reaches two conclusions. (1) With the objective of economic development, Chinese cities have consistently maintained strong connections with cities in both the developed and developing countries. (2) Chinese cities’ transnational partnerships do not observably promote decentralisation, and China’s political decentralisation is much more unstable than its economic decentralisation. Overall, both the binary partnership establishments and the dual-track decentralisation in political and economic aspects are highly embedded in China’s interstitial and transitional position as a Global East country.

## 1. Introduction

In the era of globalisation, economically and politically functional relations among cities in different countries receive increasing academic attention [1–3]. Amongst the various types of functional inter-city links, “city-to-city partnership” is one of the earliest inter-city connections but has been relatively less studied [4, 5]. Existing studies on transnational city-to-city partnerships generally focus on the staged process of partnership establishments and objectives in the geopolitical context worldwide but are hardly concerned with the partnerships’ *de facto* results, organisational structures, successful and failed factors [4, 6, 7]. Meanwhile, more recent studies on transnational city-to-city partnerships of Turkey, Morocco, and Peru repeatedly highlight that promoting decentralisation and building local governance capacity have become prioritised objectives of the city-to-city transnational partnerships mainly between developed and developing countries [5, 8]. However, considering the recent critical debates claiming that the developed-to-developing hemispheric epistemology largely overlooks the uniqueness of the transitional socialist world, studies on the city-to-city partnership establishments and objectives should be more cautious when probing into the Global East countries [9, 10]. Among the Global East countries, different from its counterparts in Eastern Europe, China exhibits more characteristically specific transnational city-to-city partnerships, as it moved away from a centrally planned economy to a transitional socialist market economy in a gradual manner via marketization, globalisation, and decentralization [11–13]. Transnational city-to-city partnerships of Chinese cities have the potential to enrich our understanding of this type of inter-city linkages of a unique Global East country. To this end, two research questions on Chinese cities need to be explicitly studied. (1) Are the Chinese cities’ transnational city-to-city partnership establishments and objectives remarkably different from the existing international body of knowledge primarily derived from the Global North and Global South countries? (2) In China, whether and how decentralisation and local governance are promoted by the transnational city-to-city partnerships?

In order to cope with the research questions, this study selects 28 Chinese world cities and quantitatively as well as qualitatively analyses the staged process of their transnational partnership establishments and objectives. It also positions the research findings into the existing international body of knowledge and seeks to extract the commonness and otherness of Chinese cities’ partnerships from global, national, and local geopolitical contexts. The remainder of this paper is arranged as follows. The introduction is followed with a literature review on the existing knowledge on transnational city-to-city partnership establishments and objectives, as well as the necessity of studying Global East countries and China in particular. Then the methodology, including study case, data collection, and analysis methods, is elaborated. The empirical section is comprised of the staged process of Chinese cities’ outward partnership establishments and objectives, with a thorough comparison of the empirical findings to the existing international body of knowledge. Finally, we discuss the findings in a wider discourse and draw conclusions.

## 2. Literature review

### 2.1 Transnational city-to-city partnerships rising as global inter-city linkages

Since the 1990s, increasingly advanced transportation, telecommunication, and information technologies have upgraded inter-city linkages from a national scale to a continental and global scale. Herein, academic focus gradually shifts to highlight the study of global inter-city networks [1, 2]. A pile of studies concentrate on how advanced producer services (e.g., finance, accountancy, law, advertisement, management consultancy, etc.) promote economic networking of global cities [14, 15]), while elevating scholarly efforts turn to transnational inter-city linkages driven by large-scale infrastructures, including telecommunication, internet, airline and marine transportation [16, 17]. In parallel, recent studies have appeared to diversify global inter-city links shaped by political, social, and cultural interactions [3, 18, 19]. Among these typologies of inter-city linkages, transnational city-to-city partnership is an earliest existed but surprisingly little studied topic [4, 7].

Transnational city-to-city partnerships, also known as “twin city” in Europe and “sister city” in North America, indicate the establishments and practices of relatively formal and long-term relationships, primarily initiated by local governments, between cities across different countries [20]. Transnational city-to-city partnership initiatives emerged at the beginning of the 20^th^ century and became proliferated after the Second World War, being conceived as political bridges to facilitate peace. Moreover, influenced by economic globalisation and geopolitical multi-polarisation, the contribution of city-to-city partnerships to benefiting one another through knowledge sharing, resource pooling, and technological joint cooperation became key objectives, which resulted in the transformation of transnational partnerships from a political peace agreement to a multi-dimensional inter-city cooperation [4]. Herein, what the objectives, results, and promoting factors of transnational city-to-city partnerships are and how they evolve in the dynamic geopolitical context attracts ascending academic concern.

### 2.2 The staged process of transnational city-to-city partnership establishments worldwide

Transnational city-to-city partnerships emerged at the beginning of the 20^th^ century with the objectives of exchanging technological knowledge and binding countries in Europe together [4]. In the early period in the aftermath of the Second World War, with the aims of building peace and reconciliation, city-to-city partnerships, firstly initiated by France and Germany and later covering other European countries, became effective and recurrent [21, 22]. Meanwhile, to prevent Western Europe from embracing communism, the United States (US) also stimulated establishing “sister city” relations in the 1950s and 1960s [4, 21]. In 1948 the Marshall Plan was approved by the US Congress and thus over 12 billion dollars were loaned by the US government to Western Europe. In this matter, the trans-continental economic assistance channelled closer ties between the two sides of North Atlantic, so transnational city-to-city partnerships were established between the US and Western-European countries including the UK, France, and Western Germany by the 1960s [23].

After rapid economic growth in the 1960s, the productivity and market became gradually saturated in the US and Western European countries. Meanwhile, an increasing number of countries in Asia and Africa began to strive for sovereignty independence from their suzerains. Hence, after losing the colonial control on former colonies, Western European countries, especially the UK and France, started to establish, develop, and maintain economic colonial relations with the new sovereign countries so as to import inexpensive raw materials and resources, to export domestic surplus industrial products, and to use the economic colonies’ low-cost labour forces. Economically dependent relations have replaced the former politically associated relations between developed countries and their former colonies, such as Singapore, Malaysia, Indonesia, Philippines, and Thailand that experienced rapid industrialisation in the 1970s. Therefore, from the 1970s to the early 1980s, city-to-city partnerships primarily connected former colonists and the new sovereign countries [24].

In the late 1980s, the political institution, economic system, and social ideology of Eastern European countries drastically shifted from Soviet communism to capitalism, and intimate ties between cities in Western and the transitional Eastern Europe, such as Western Germany and Poland, were established with the objectives of solidarity, cultural exchange, altruism, and countering the Cold War [4, 21, 25]. Meanwhile, influenced by neoliberalism since the 1980s, cities in some developing countries began to endeavour to the decentralisation of the national institutional system, strengthened local government authorities, and capacity building of the civil society [4]. However, the decentralisation process was obstructed by the state via financial resource control, among other ways. For instance, in Peru, the state started to delegate some administrative functions to local governments in the 1980s but still occupied a predominant role in managing local financial resources, so in the 1990s the vulnerable decentralisation of Peru was abruptly stopped by President Fujimori, and all sub-national institutes were suddenly abolished [4]. In a similar vein, since the 1990s Turkey has established a dual local governance system consisted of a local-elected mayor and a state-appointed officer, both of which work in parallel to deal with local administrative affairs. However, in this dual-governance system, the elected mayor’s mandates are still strongly tied to state government policies due to the lack of independent fiscal management power. In Morocco, even though the state has already implemented political decentralisation in the 1960s, up till the 1990s the financial resources of local governments were still manipulated by state financial departments [21]. The insufficient decentralisation in Peru, Turkey, and Morocco denotes that decentralisation is rather a progressive process, including different stages such as de-concentration, divestment, delegation, and devolution, and most of the state bodies only de-concentrate some daily executive works to local governments but hardly divest financial resources or delegate central authorities to the local level [21]. Therefore, through establishing city-to-city partnerships with developed European countries whose local governance and civil society are much more mature, cities in developing countries to some extent achieved devolution and gained local government authority. For instance, the LOGO South program set up by the Dutch government has transferred some policy and knowledge related to finance, water, and waste management to local governments in Benin, South Africa, Indonesia, and Nicaragua, and the four developing countries’ local governance capacity has been significantly strengthened [26]. In this sense, comparing to the city-to-city partnerships between developed and developing countries at the previous stage, since the late 1980s they presented two subtle differences. First, the involved countries geographically expanded to former socialist members in Eastern Europe, moving beyond the scope of capitalist economies. Second, and more distinctively, the objective of city-to-city partnerships shifted from economic cooperation to strengthening local governance capacity.

Since the end of the 20^th^ century, sustainable development receives attention worldwide, and some social and cultural issues including environment protection, anti-virus, poverty alleviation, and immigrant integration have become globally concerned topics. Under this circumstance, city-to-city partnerships between developed and developing countries become more reciprocal [27]. A bunch of case studies indicate that cities in Turkey and Morocco can empower local governance, build civil society, and raise administrative efficiency through learning knowledge from their partners in Netherlands, thus Dutch cities can as well know more about the ethnic cultures and social behaviours of Turkish and Moroccans so as to alleviate ethnic conflicts and to promote social integration of Turkish and Moroccan immigrants into Netherlands [21, 26]. Meanwhile, due to the multilateral political and economic organisation initiated by rising Brazil-Russia-India-China-South Africa (BRICS), exemplified as Commonwealth of Independent States established in 1993, Shanghai Cooperation Organisation in 2001, and the African Union in 2002, city-to-city partnerships across developing countries are playing an increasingly pivotal role in forming transnational intercity linkages [5, 23, 28]. Consequently, transnational city-to-city partnerships in the 21^st^ century present two presumable features. First, the objectives of partnerships are more concerned with social and cultural issues. Second, transnational city-to-city partnerships between developing countries are rapidly growing prominence.

### 2.3 Increasing attention on transnational city-to-city partnerships within Global East countries

Previous studies have elucidated the staged process of transnational city-to-city partnership establishments and objectives from a dynamic geopolitical perspective. However, this process is criticised for overlooking the meticulous differences of cities in the Global East countries [9]. Global East involves the transitional socialist countries which occupy an interstitial position between developed and developing countries in economic, political, and geopolitical aspects [10]. First, Global East countries, with their relatively higher proportion of heavy industries and stronger domestic industrial bases, may to some extent deviate from being perceived as developing countries, and in the meantime they can hardly be categorised into developed economies due to their less competitive innovation capacity and service industries. Second, Global East countries, generally as the so-called Second World during the Cold War period, have been experiencing institutional reforms from centrally planned socialism to market-oriented economies since the 1980s, and most Global East countries have neither been entirely colonised nor completely retained independent sovereignty in the pre-modern history. Third, Global East countries are generally situated in the geopolitical semi-core and semi-periphery areas in the Second World, and the core-periphery geopolitical structure has been drastically changing over the recent years. Some Central Asian, South Caucasus, and Southeast European countries, perceived as the former semi-core countries such as Kazakhstan, Georgia, and Yugoslavia, plausibly migrate to more peripheral positions in the system, while China and some Eastern European countries such as Poland are economically approaching to the core group.

However, extant studies are generally inclined to regard the Global East countries as identical to developing countries, and existing knowledge on transnational city-to-city partnerships overlooks Global East countries’ uniqueness in political, economic, and most importantly geopolitical aspects [10]. Meanwhile, without the consideration of Hong Kong, Macau, and Taiwan, China is a Global East country, combining the characteristics of both developed and developing countries. From the 1950s to 1970s, mainland China underwent an intensively planned and highly centralised development paradigm, following the institutional and social development pattern of the Soviet Union. Since the 1980s, mainland China began to transform toward a market-oriented economy, and the domestic political, economic, socio-cultural as well as geopolitical contexts are in all-sided transition [13]. Thus, regarding the priority of local governance in existing studies and the transitional nature of the Global East countries, two emerging questions call for further explicit exploration on Chinese cities: (1) Are Chinese cities’ transnational city-to-city partnership establishments and objectives significantly different to the existing international body of knowledge? (2) In China, whether and how decentralisation and local governance are promoted by the transnational city-to-city partnerships?

## 3. Methodology

To answer the above two research questions, we select 28 Chinese world cities to analyse their establishments and objectives of transnational city-to-city partnerships and further compare the findings to the existing international body of knowledge.

### 3.1 Research case and data collection

With the rapid process of globalisation, Chinese cities become more proactive in embracing transnational city-to-city partnerships in order to attract investment, production resources, and knowledge. Up till 2016, the Globalisation and World Cities Research Network (GaWC), an authoritative academic organisation of world city network studies, has listed 28 mainland Chinese cities as world cities to acknowledge their fruitful transnational cooperation and international influences (Table 1). Meanwhile, these listed 28 cities, all of which have become national and regional economic centres after mainland China implemented the economic reform and opening up policy, are also the most successful cities achieving market-oriented transformation. In this matter, to underline the attributes of China’s transition from a planning-centred to a market-oriented economy, we selected the 28 mainland Chinese cities as our research cases.

**Table 1:** Number of partner cities and their origins.

Rank	Chinese cities	City	Country
Alpha+, 9^th^	Shanghai	86	60
Alpha+, 6^th^	Beijing	73	53
Gamma, 163^rd^	Chongqing	46	32
Sufficiency, 340^th^	Harbin	36	27
Alpha-, 40^th^	Guangzhou	36	33
Gamma-, 198^th^	Suzhou	37	24
Beta-, 100^th^	Chengdu	36	30
Gamma+, 140^th^	Hangzhou	32	26
Gamma-, 209^th^	Xi’an	34	28
Beta-, 113^th^	Tianjin	28	21
Gamma+, 143^rd^	Qingdao	27	24
Gamma-, 190^th^	Wuhan	28	25
High Sufficiency, 221^st^	Jinan	27	26
Sufficiency, 257^th^	Kunming	24	21
Beta, 85^th^	Shenzhen	23	23
Sufficiency, 305^th^	Changchun	20	19
Gamma, 171^st^	Xiamen	20	19
Gamma-, 213^th^	Shenyang	18	13
Gamma+, 139^th^	Nanjing	23	23
Sufficiency, 333^rd^	Nanning	19	19
Sufficiency, 267^th^	Fuzhou	17	14
Sufficiency, 349^th^	Urumqi	16	13
Sufficiency, 332^nd^	Zhengzhou	16	15
Gamma, 160^th^	Dalian	11	8
Sufficiency, 311^th^	Hefei	12	12
Sufficiency, 321^st^	Ningbo	11	11
Gamma-, 201^st^	Changsha	12	9
Sufficiency, 294^th^	Taiyuan	12	11

Meanwhile, aiming at analysing the staged process of Chinese cities’ city-to-city partnership establishments and objectives, the basic information of research cases’ transnational partner cities, including the names, countries, and the year of partnership establishment are sourced from the official website of Chinese International Friendship Cities Association (CIFCA). The xGeocoding software is utilised to collect all partner cities’ geographical coordinates.

### 3.2 Methods

Since the founding of the People’s Republic of China, Chinese Communist Party (CCP) has been playing a dominant role in almost all aspects of China’s development such as announcing economic policies, issuing development outlines, stimulating the transition toward market-oriented socialism, and implementing taxation reforms. Hence, considering the influence of the national institutional context on Chinese cities’ transnational partnerships, we choose to divide the entire partnership formation process, starting from 1973 to 2019, with nearly a five-year interval, which is rather consistent with the five-year tenure of CCP. Considering COVID-19’s abruptly negative influence on interrupting international communications to an unnormal level, our data collection ends in 2019 and therefore primarily applies to the pre-pandemic world. Second, we use ArcMap to present the geographical locations of all city-to-city partnerships at each stage, aiming at delineating the geographical pattern of Chinese cities’ partnership establishments. By deploying the social network analysis(SNA), UCINET 6.0 is used to present the number of partner cities as well as the frequency of partnership ties by exhibiting different size of nodes and different width of lines. Third, we compare the staged process of Chinese cities’ partnership establishments to the existing international body of knowledge, and refer to global, national, and local geopolitical discourses to examine Chinese cities’ dynamic partnership objectives. The distinctive characteristics of Chinese cities’ partnership formation process is also summed up.

## 4. Results

### 4.1 General pattern of Chinese cities’ transnational partnerships

Up till the end of 2019, there are 780 cities in total from 115 countries officially establishing partnerships with the 28 selected Chinese world cities. Shanghai establishes transnational partnerships with 86 cities from 60 countries, but Dalian only links with 11 cities from 8 countries, implying a disproportionate distribution of partnership establishments among Chinese world cities. The correlation between the GaWC world city ranking and city-to-city partnerships is unobvious, implying that the prioritised factors of city-to-city partnerships may be different with the world city ranking. The latter specifically focuses on advanced producer service. Shenzhen, ranking 85^th^ at Beta level, has fewer partner cities than its Gamma counterparts (Table 1).

### 4.2 Staged process of Chinese cities’ transnational partnership establishments and objectives

From 1973 to 2019, Chinese cities exhibited a five-stage process of transnational partnership establishments.

#### (1)1973-1977: Peace building and diplomacy

**Fig 1:**
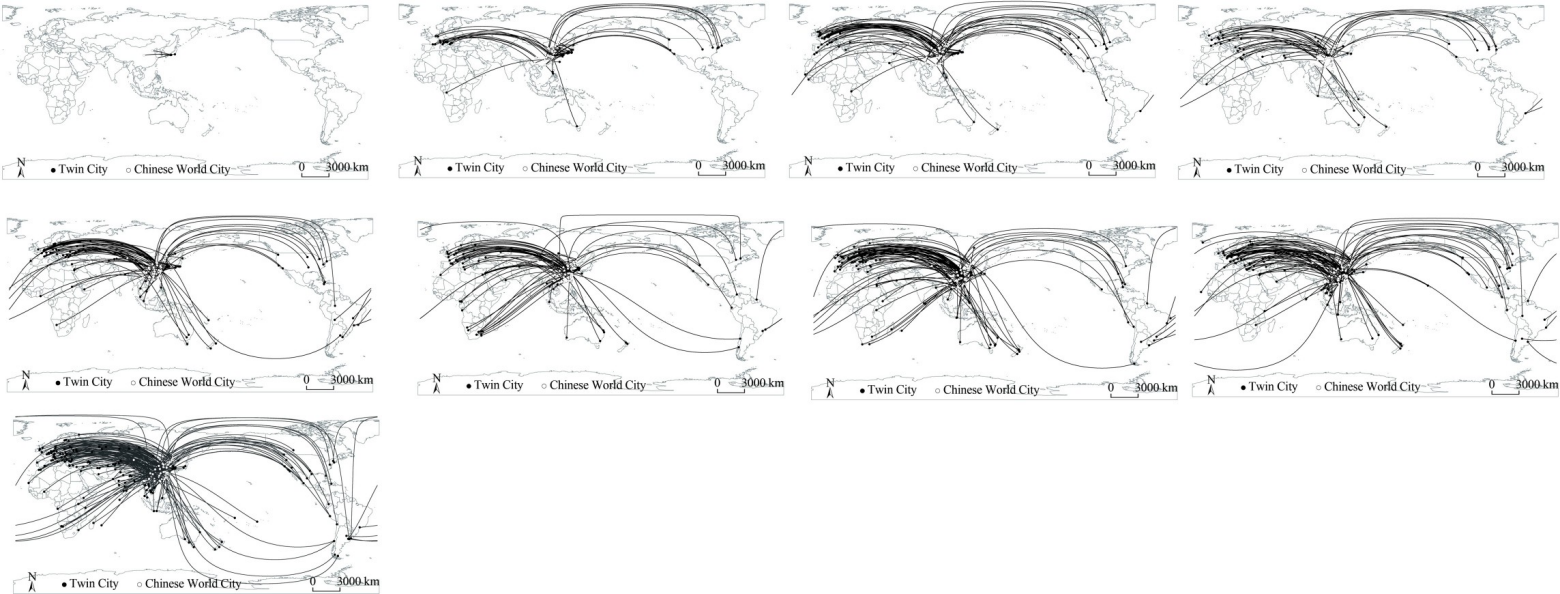
The establishments of Chinese cities’ transnational partnership. (a)1973-1977, (b)1978-1982, (c)1983-1987, (d)1988-1992, (e)1993-1997, (f)1998-2002, (g)2003-2007, (h)2008-2012, (i)2013-2019. Note: BJ Beijing; CC Changchun; CS Changsha; CD Chengdu; CQ Chongqing; DL Dalian; FZ Fuzhou; GZ Guangzhou; HZ Hangzhou; HB Harbin; HF Hefei; JN Jinan; KM Kunming; NJ Nanjing; NN Nanning; NB Ningbo; QD Qingdao; SH Shanghai; SY Shenyang; SZ Shenzhen; SU Suzhou; TY Taiyuan; TJ Tianjin; UR Urumqi; WH Wuhan; XA Xi’an; XM Xiamen; ZZ Zhengzhou; AFG Afghanistan; ALB Albania; ARE United Arab Emirates; ARG Argentina; ARM Armenia; AUS Australia; AUT Austria; BDI Burundi; BEL Belgium; BFA Burkina Faso; BGD Bangladesh; BGR Bulgaria; BIH Bosnia and Herzegovina; BLR Belarus; BOL Bolivia; BRA Brazil; BRN Brunei; CAN Canada; CHE Switzerland; CHL Chile; CIV Côte d’Ivoire; CMR Cameroon; COG Congo; COL Colombia; CPV Cape Verde; CRI Costa Rica; CUB Cuba; CYP Cyprus; CZE Czech; DEU Germany; DNK Denmark; ECU Ecuador; EGY Egypt; ESP Spain; ETH Ethiopia; FIN Finland; FJI Fiji; FRA France; GBR the United Kingdom; GEO Georgia; GMB Gambia; GRC Greece; GUY Guyana; HRV Croatia; HUN Hungary; IDN Indonesia; IND India; IRL Ireland; IRN Iran; ISL Iceland; ISR Israel; ITA Italy; JAM Jamaica; JOR Jordan; JPN Japan; KAZ Kazakhstan; KEN Kenya; KGZ Kyrgyzstan; KHM Cambodia; KOR South Korea; LAO Laos; LKA Sri Lanka; LTU Lithuania; LVA Latvia; MAR Morocco; MDG Madagascar; MDV Maldives; MEX Mexico; MLT Malta; MMR Myanmar; MNE Montenegro; MNG Mongolia; MOZ Mozambique; MRT Mauritania; MUS Mauritius; MYS Malaysia; NAM Namibia; NLD Netherlands; NOR Norway; NPL Nepal; NZL New Zealand; PAK Pakistan; PER Peru; PHL Philippines; PNG Papua New Guinea; POL Poland; PRK North Korea; PRT Portugal; QAT Qatar; ROU Romania; RUS Russia; SDN Sudan; SGP Singapore; SLE Sierra Leone; SRB Serbia; SUR Suriname; SVK Slovakia; SVN Slovenia; SWE Sweden; TGO Togo; THA Thailand; TJK Tajikistan; TKM Turkmenistan; TUR Turkey; UGA Uganda; UKR Ukraine; URY Uruguay; USA the United States; UZB Uzbekistan; VNM Vietnam; VUT Vanuatu; WSM Samoa; YEM Yemen; ZAF South Africa; ZWE Zimbabwe.

**Fig 2:**
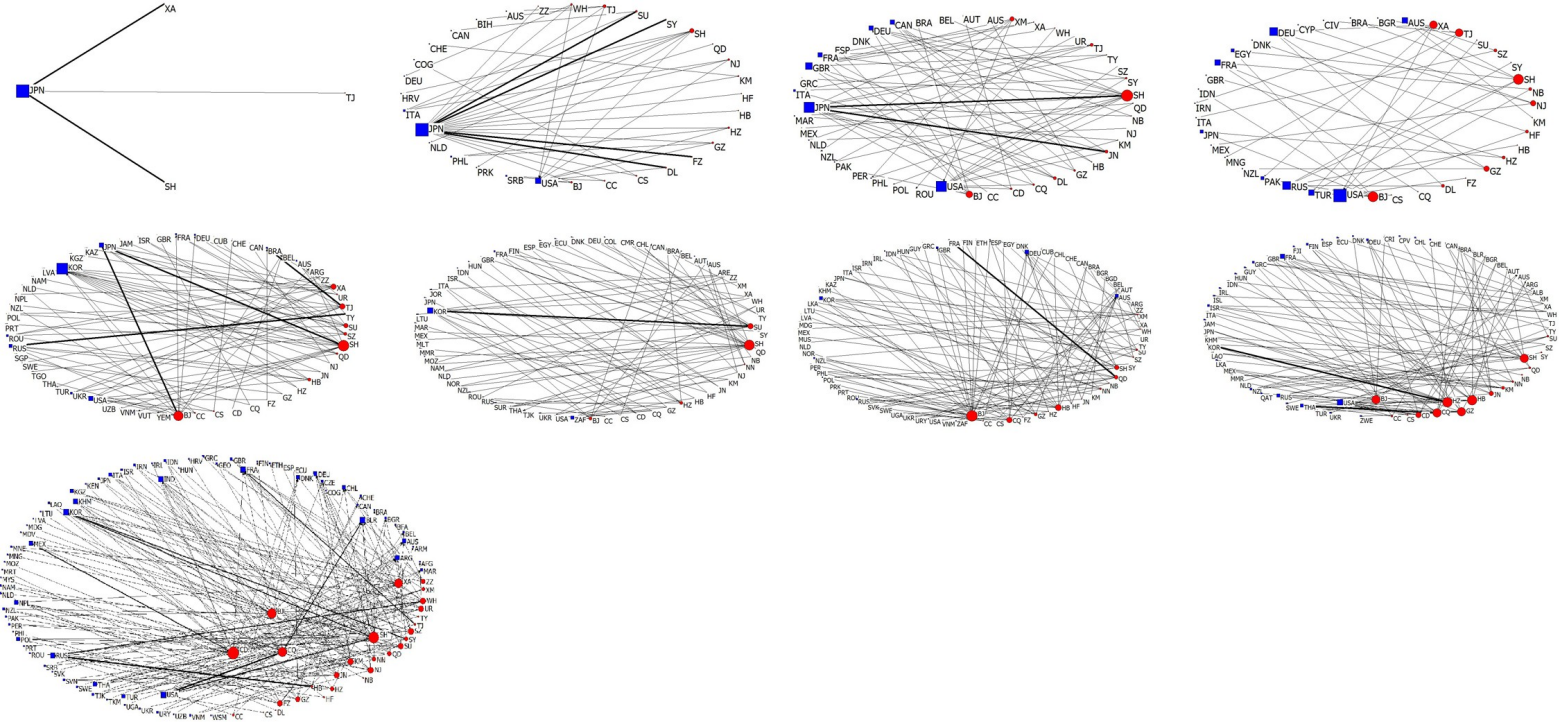
The Chinese cities’ transnational partnership network. (a)1973-1977, (b)1978-1982, (c)1983-1987, (d)1988-1992, (e)1993-1997, (f)1998-2002, (g)2003-2007, (h)2008-2012, (i)2013-2019. Note: BJ Beijing; CC Changchun; CS Changsha; CD Chengdu; CQ Chongqing; DL Dalian; FZ Fuzhou; GZ Guangzhou; HZ Hangzhou; HB Harbin; HF Hefei; JN Jinan; KM Kunming; NJ Nanjing; NN Nanning; NB Ningbo; QD Qingdao; SH Shanghai; SY Shenyang; SZ Shenzhen; SU Suzhou; TY Taiyuan; TJ Tianjin; UR Urumqi; WH Wuhan; XA Xi’an; XM Xiamen; ZZ Zhengzhou; AFG Afghanistan; ALB Albania; ARE United Arab Emirates; ARG Argentina; ARM Armenia; AUS Australia; AUT Austria; BDI Burundi; BEL Belgium; BFA Burkina Faso; BGD Bangladesh; BGR Bulgaria; BIH Bosnia and Herzegovina; BLR Belarus; BOL Bolivia; BRA Brazil; BRN Brunei; CAN Canada; CHE Switzerland; CHL Chile; CIV Côte d’Ivoire; CMR Cameroon; COG Congo; COL Colombia; CPV Cape Verde; CRI Costa Rica; CUB Cuba; CYP Cyprus; CZE Czech; DEU Germany; DNK Denmark; ECU Ecuador; EGY Egypt; ESP Spain; ETH Ethiopia; FIN Finland; FJI Fiji; FRA France; GBR the United Kingdom; GEO Georgia; GMB Gambia; GRC Greece; GUY Guyana; HRV Croatia; HUN Hungary; IDN Indonesia; IND India; IRL Ireland; IRN Iran; ISL Iceland; ISR Israel; ITA Italy; JAM Jamaica; JOR Jordan; JPN Japan; KAZ Kazakhstan; KEN Kenya; KGZ Kyrgyzstan; KHM Cambodia; KOR South Korea; LAO Laos; LKA Sri Lanka; LTU Lithuania; LVA Latvia; MAR Morocco; MDG Madagascar; MDV Maldives; MEX Mexico; MLT Malta; MMR Myanmar; MNE Montenegro; MNG Mongolia; MOZ Mozambique; MRT Mauritania; MUS Mauritius; MYS Malaysia; NAM Namibia; NLD Netherlands; NOR Norway; NPL Nepal; NZL New Zealand; PAK Pakistan; PER Peru; PHL Philippines; PNG Papua New Guinea; POL Poland; PRK North Korea; PRT Portugal; QAT Qatar; ROU Romania; RUS Russia; SDN Sudan; SGP Singapore; SLE Sierra Leone; SRB Serbia; SUR Suriname; SVK Slovakia; SVN Slovenia; SWE Sweden; TGO Togo; THA Thailand; TJK Tajikistan; TKM Turkmenistan; TUR Turkey; UGA Uganda; UKR Ukraine; URY Uruguay; USA the United States; UZB Uzbekistan; VNM Vietnam; VUT Vanuatu; WSM Samoa; YEM Yemen; ZAF South Africa; ZWE Zimbabwe.

The People’s Republic of China was founded in 1949. At the beginning of the 1950s, China built close diplomatic relations with the Soviet Union and certain Eastern European countries. However, the transient coalition between China and the Soviet Union split since 1960, and the economic development of mainland China became unrest due to the domestic political movements and military conflicts at border areas. In 1972, the US President Nixon officially visited China, for rebuilding the diplomatic communications between China and the Western capitalist world. Among the capitalist economies, Japan enjoys the closest political, economic, and cultural relations with China, but intensive military conflicts between China and Japan unfortunately broke out during the Second World War. Therefore, with the objectives of peace building and reconciliation, as well as rebuilding the diplomatic relations to stimulate further co-development, Shanghai, Tianjin, and Xi’an initially established city-to-city partnerships with five Japanese cities. At this stage, Japan was the only country possessing transnational partnerships with the selected Chinese cities (Figs 1A and 2A).

#### (2)1978-1987: Economic growth and decentralisation

In the late 1960s, in order to export surplus industry products as well as excavate raw materials and resources, the developed world energetically invested in newly sovereign countries, so that some countries and regions in Asia, typically exemplified as the Four Asian Dragons (i.e., South Korea, Taiwan, Hong Kong, and Singapore), stepped into miraculous economic growth and high-speed industrialisation since then. Unexpectedly, at the end of the 1970s, due to the rapidly increasing labour and land cost, a bunch of traditional industries (e.g., textiles, electronic products, toys) in Japan and the Four Asian Dragons started to seek other places for industrial relocation. At the moment, the Chinese central government officially announced to implement the economic reform and opening up policy, and the price for raw materials, labour forces and land were quite cheap in the coastal regions, so local governments there started to entrepreneurially attract foreign investments from industrialised countries and regions with geographical proximity. As the Chinese central government did not have sufficient resources to invest and stimulate indigenous industrialisation, local governments’ entrepreneurial behaviours were tacitly endorsed by higher-level authorities [12]. Thus, the acquiescence of the central government inadvertently stimulated plausible and vulnerable decentralisation in administrative and economic aspects in the late 1980s.

Hence, at this stage, Chinese cities still mainly established transnational city-to-city partnerships with Japanese cities, with Dalian, Shenyang, Suzhou, Fuzhou, Jinan, and Shanghai having the most frequency of these Sino-Japanese relations (Figs 1B, 1C and 2B and 2C). Dalian and Shenyang are located in Liaoning Province in the Northeast of China, where was colonised by Japan for almost forty years after the Russo-Japanese War during 1904–1905. Shanghai, Suzhou, and Fuzhou were forced to open as trading ports after China lost wars against the UK and Japan in the pre-modern age. Moreover, Shanghai and Suzhou in the Yangtze River Delta, where merchant ships from Kyushu of Japan could directly reach and unload commercial products. Fuzhou and Jinan are adjacent to Taiwan and the Korean Peninsula where were colonised by Japanese governments after China lost the 1^st^ Sino-Japanese War in 1895 and reluctantly signed the Treaty of Shimonoseki. Hence, the six Chinese cities with the most frequent Sino-Japanese partnerships are all formerly colonised by Japan or adjacent to Japan colonies, implying consistencies with the existing knowledge that partnership establishments in the 1970s generally emerged between former colonists and newly sovereign countries. Moreover, some Chinese cities also established city-to-city partnerships with the US, Canada, and some other Western countries, including the UK, Germany, and France, primarily with the aims of attracting foreign investment to promote local industrialisation (Fig 2B and 2C).

#### (3)1988-1992: Economic cooperation and political anti-decentralisation

After rapid industrialisation in the 1980s, Chinese local governments started to manage to attract heavy industries (e.g., automobile, petrochemical) and knowledge-intensive industries (e.g., bio-pharmaceutics, computer science), aiming at improving economic efficiency and striving for a more equivalent status to Japan and the Four Asian Dragons in global value allocation [29]. Therefore, the priority of city-to-city partnerships at this stage has shifted from economic growth towards economic cooperation, and the US, Germany, Australia, Turkey, and France became the major partnership destination countries. Meanwhile, although the frequency of Sino-Russian relations increased significantly, the rise of the city-to-city partnerships between China and other Eastern European countries was not as substantial as the increase of partnerships between the Western capitalist economies and post-socialist Eastern Europe countries (Figs 1D and 2D). And the establishments of partnerships between China and post-socialist Eastern Europe mainly aimed at economic affairs, such as facilitating potential cross-border trade, international tourism, and resource exploitation, instead of solidarity and altruism [24, 30]. This inconsistency may be explained by the similar ideology of China and Eastern Europe, as they both belonged to the socialist group, and thus their diplomatic, economic, and cultural communications were not much obstructed during the Cold War.

Meanwhile, at this stage decentralisation was suddenly stopped. After the decentralised reforms lasting almost ten years, China was standing at the intersection of state power decentralisation and ideological transition in the late 1980s, and therefore some social requests asking for drastically further political reforms emerged. However, China has never abandoned socialism but just tactfully creates a political ideology termed “Socialism with Chinese Characteristics” to define China’s market-oriented socialism, so the social requests for ideological abandonment alerted state government to strengthen its dominant status in political power [31, 32]. In this regard, economic decentralisation not only failed in resonating with political decentralisations but also was faced with serious challenges and opposition from the state government.

#### (4)1993-2007: Improving local competitiveness and re-decentralisation

In the 1990s, the Chinese central government faced up with increasingly serious financial deficit, so in 1994 the central government officially promulgated the institutional reforms of taxation [33]. In this round of reform, the central government devolved certain fiscal expenses including administration, audition, armed forces, infrastructure investment and maintenance, R&D of local enterprises as well as commercial, cultural, educational, and health business expenses, to local governments. On the one hand, it immediately reduced the central government’s fiscal expenditure, while on the other hand it increased the financial burden of local governments. Moreover, China’s accession to the World Trade Organisation in 2001 symbolised the disappearance of tariff protection, consequently exposing Chinese local enterprises to global fierce competition. Nevertheless, devolving fiscal expenditures inevitably led to delegating the administrative power of tax collection, and the taxation reform also allowed local governments to independently collect tax on local enterprises, property, agricultural activities, and land-added values. Therefore, in order to improve local enterprises’ competitiveness to increase local governments’ fiscal revenue, major Chinese cities (e.g., Shanghai, Beijing, Suzhou, Qingdao) actively established city-to-city partnerships with a larger number of cities in developed countries (e.g., the US, Japan, Germany, and Australia) and used the collected tax to fund local enterprises to better learn advanced knowledge of production and management from companies in partner cities [34] (Fig 1E–1G). At the moment, China and South Korea officially established diplomatic relations in 1992. Hence, the disappearance of bilateral diplomatic barriers has significantly stimulated the city-level partnerships between China and South Korea (Fig 2E-2G). Therefore, the devolution of fiscal expenditures and the delegation of taxation power implied a re-decentralisation of the financial institution and local economic governance right after a short-term anti-decentralisation. However, two differences should be paid attention. First, this re-decentralisation resulted from a state-launched taxation reform, indicating that the re-decentralisation was vulnerable and predominated by the state. Second, instead of promoting decentralisation, transnational city-to-city partnerships were triggered, or more precisely, were guaranteed by institutional re-decentralisation.

#### (5) Since 2008: Recentralisation and strengthening worldwide influence

In 2008, the global financial crisis swept the world and heavily blew the economy of the US, European Union, and Japan. To lower the unemployment rates, governments in developed countries encouraged their domestic enterprises to withdraw the overseas investment and to a greater extent hire the local unemployed labour forces. Also, these governments stimulated residents to consume domestic products instead of the products imported from the developing world. Influenced by capital withdrawal and export shrinkage, China, the “world factory”, seriously suffered from economic decline. To avoid the further sprawl of financial crisis, the Chinese central government began to seek for new trade partners beyond the developed capitalist world. In 2013, the Chinese state initiated the Belt and Road Initiative (BRI) to establish multilateral partnerships with 126 countries and 29 international organisations. In 2015, under the promotion of the Chinese state, Asian Infrastructure Investment Bank (AIIB) was established. Up until now, AIIB already has 70 members originating from Asia, Europe, and Australia as well as 27 prospective members from Canada, South America, and North Africa. Building up trans-continental trade networks not only provides Chinese enterprises with new market niches but also enhances China’s proactive influences on the international economy [28, 35]. Thus, being consistent with the state government’s diplomatic strategies, at this stage, transnational city-to-city partnerships between China and developing countries significantly gained momentum. From 2008 to 2019, although cities in the US, France, and South Korea still occupied a substantial proportion of all newly established partner cities, the number of partner cities originating from Indonesia, Thailand, Nepal, Turkey, Russia, Belarus, Argentina, and Mexico witnessed rapid expansion (Figs 1H, 1I and 2H and 2I). However, the increase and expansion of Chinese cities’ transnational partnerships have not promoted further decentralisation of state power. Oppositely, in the eyes of state governments, decentralising financial resources and administrative power to local governments led to excessive partnerships with developed countries [36]. Once a financial tsunami broke out in the developed capitalist world, China could hardly escape from the financial crisis and inevitably suffered from the serious consequence of economic decline. Therefore, recentralising fiscal and administrative power is necessary. The independence of local tax collection system was consequently abolished in 2018, and the state government has recaptured more financial resources from local governments. As the implementation of BRI and AIIB spends countless money, under the target of improving China’s influence on the world economy, the Chinese central government recentralised more financial resources and political power from local governments, and local governance capacity is expected to be further eroded in the future.

### 4.3 The distinctions of Chinese cities’ transnational partnership establishments and objectives compared to the existing international body of knowledge

Compared to the existing international body of knowledge of transnational city-to-city partnerships, the transnational partnerships of China as a Global East country presents distinctive features in three aspects including establishment, objective, and local governance.

In the regard of partnership establishment, Chinese cities mainly established city-to-city partnerships with cities in developed countries in East Asia, North America, and Western Europe in the past around forty years. However, three distinctions need to be elucidated (Table 2). First, after the People’s Republic of China was founded, China has deeply immersed in wars from the 1950s to 1960s at Taiwan Strait (1949–1965), in Korean Peninsula (1950–1953), Vietnam (1955–1975), Sino-Indian border (1962), and Sino-Soviet border (1969), so that China’s diplomatic situation was unrest until the 1970s. Therefore, the starting point of Chinese cities’ transnational partnerships lagged nearly twenty years behind the global connections. Second, in the first 15 years, due to the frequent Sino-Japanese communications in the historical period, Chinese cities mainly established partnerships with cities in Japan, instead of cities in the US or other Western European countries. Third, in the early 1990s, the elevation of partnership establishments between China and the former socialist Europe was not as significant as the increase between Western and Eastern Europe, mainly due to China’s socialism ideology during the Cold War period.

**Table 2:** Comparison of Chinese cities’ transnational partnerships and the existing international body of knowledge.

Existing Knowledge			The Selected 28 Chinese Cities			
Stage	Establishment	Objective	Stage	Establishment	Objective	Local Governance
1950s-1960s	Within Europe	• Peace and Reconciliation				
	U.S.-Western Europe	• Economic Assistance • Communism Prevention				
1970s-Early 1980s	Colonist-New Sovereignty	• Economic Colonisation	1973–1977	Japan	Peace and Diplomacy	
			1978–1987	Japan, U.S., Canada, U.K., Germany, France	Economic Growth	Decentralisation
Late 1980s-1990s	West-Socialist East	• Solidarity • Cultural Exchange • Protest against Cold War				
			1988–1992	Russia, U.S., Germany, Australia, Turkey, France	Economic Cooperation	Administrative Anti-decentralisation
	Developing-Developed	• Local Governance	1993–2007	South Korea, U.S., Japan, Germany, Australia	Competitiveness Improvement	Re-decentralisation
Since the end of the 1990s	Developing-Developed Within Developing Countries	• Environment Protection • Anti-Virus • Poverty Alleviation • Immigrant Integration • Local Governance				
			Since 2008	Developed: U.S., France, South Korea; Developing: Indonesia, Thailand, Nepal, Turkey, Russia, Belarus, Argentina, Mexico	Strengthening Influence on Global Economy	Recentralisation

Moreover, Chinese cities’ partnership objectives are distinguished from its Western counterparts (Table 2). Existing knowledge highlights that the objectives of the transnational city-to-city partnerships have become increasingly multi-dimensional. In the early stage, the objectives of transnational partnerships are confined in politics and diplomacy, such as peace building and bi-lateral reconciliation. Even the economic assistance of the US to Western Europe was highly related to the prevention of spreading communism. From the 1970s to early 1980s, economic colonisation became more prioritised. At the end of the 1980s, solidarity and cultural exchange were highlighted between Western and Eastern Europe where were divided for a long term. During this time period, economic and political decentralisation and local governance also attracted more attention. Since the new century, more and more social issues, including environment protection, anti-virus, poverty alleviation, and immigrant integration, have been incorporated into partnership objectives. Meanwhile, local governance and civil society building have become more prioritised under the promotion of increasingly frequent transnational communications. By contrast, the objectives of Chinese cities were distinctively related to economic development except for peace building and diplomacy in the 1970s in a short term. Comparing the subtle differences among economic growth, economic cooperation, competitiveness enhancement, and strengthening international influences at different stages, Chinese cities’ transnational partnership objectives switched from economic growth to economic independence and finally to economic influences, implying an ambitious objective for economic significance in the future.

Last but not least, Chinese cities’ transnational partnerships have unstably promoted local governance and decentralisation, presenting three distinctive characteristics that could be further discussed. First, the decentralisation of Chinese cities includes the economic and administrative aspects, while the process of administrative decentralisation is much more unstable than economic decentralisation. In the past forty years, economic decentralisation has overall been moving forward. However, administrative decentralisation has experienced decentralisation, anti-decentralisation, re-decentralisation, and recentralisation, exhibiting repeated conversions among centralisation, decentralisation, and recentralization. The progressive economic decentralisation and unstable administrative decentralisation exactly reflects the transitional characteristics of China as a Global East country—circumscribing market-oriented reforms in economic domains and continually pursuing socialism in political affairs [31]. Second, economic decentralisation is a strategy implemented by the Chinese state to stimulate economic growth rather than an outcome of city-to-city transnational partnerships, and the state has always maintained a decisive status in promoting or prohibiting decentralisation [32]. When the state did not have adequate resources to invest in infrastructures and stimulate top-down massive industrialisation in the 1980s, both economic and political power were decentralised to local governments. In the 1990s when the state government faced up with potentially serious fiscal deficit, economic decentralisation was encouraged again. Third, the administrative decentralisation is mobilised to be completely consistent with the state government’s political interests, and once a choice is required to be made between economic and political interests, the state government would choose the latter without any hesitation. For instance, at the end of the 1980s, once the economic decentralisation might be destructive to the political institution, an anti-decentralisation initiative instantly spread and economic decentralisation was subsequently rethought.

## 5. Conclusions and discussion

In this article, we quantitatively analysed the staged process of 28 Chinese world cities’ transnational partnership establishments and objectives and further discussed the relationships between partnership establishments and local governance. On the basis of the empirical results elaborated, two conclusions can be drawn to answer the two research questions.

First, with the Global East characteristics, Chinese cities’ transnational partnerships present distinctive characteristics to the existing knowledge. In the aspect of partnership establishments, Chinese cities maintained relatively strong connections with the majority of developed countries in North America (i.e., the US), Western Europe (i.e., Germany, France) and East Asia (i.e., Japan). Recently, Chinese cities increasingly established partnerships with cities in developing countries. In the aspect of partnership objectives, compared to the multi-dimensional objectives in the existing knowledge, Chinese cities’ partnership objectives distinctively focused on economic development, presenting a progressive process from economic growth to economic independence, and finally to strengthening economic international influences. Both the establishments and objectives implied Chinese cities’ binary partnerships with developed and developing countries, which is consistent with China’s interstitial economic situation as a Global East country [10].

Second, being distinctive in city-to-city partnerships’ promotion on decentralisation and local governance, Chinese cities’ transnational partnerships did not exert remarkably positive effects on national decentralisation. Although a continuous increase of the number of partner cities and a rapid expansion of partnership establishments appeared in the past forty years, the process of China’s political decentralisation is much more unstable than its economic decentralisation, and the state has always maintained a dominant role in conditioning decentralisation process. The dual-track process, comprising unstable political decentralisation and ongoing economic decentralisation, indicates that China has been implementing a dual-track institutional reform which could be summarised as “limiting market-oriented reforms in the economic domain and continually pursuing socialism in political affairs” in its specific Global East transitional context.

This study offers supporting evidence of China, a Global East country, to answer the two research questions and to some extent encourages more open-minded thinking in social sciences [9]. Meanwhile, since some other Global East countries in transitional contexts are also confronted with severe conflicts between domestic political and economic reforms, the above two conclusions also indicate that perhaps prioritising economic development and decentralisation in the first place and promoting administrative decentralisation later on at a more appropriate time might be an ideal solution to deal with the conflicts of Global East countries’ domestic economic and political reforms. However, this study is still limited in discussing the knowledge of partnership’s establishments and objectives, but the organisational structure, success factors, and weakness points of cities’ transnational partnerships originated from Global East countries are not explicitly examined. Moreover, even though in the group of the Global East countries, China is still more or less peculiar, and thus more studies on Eastern-European and Middle-Asian countries are needed [37]. Therefore, two more issues are calling for further exploration.

First, case studies on the US-to-South Africa, Netherlands-to-Nicaragua, the US-to-Mexico denote that the maturity of civil society, eagerness of public participation, transaction cost of bilateral trade, and administrative efficiency of local governments are all deem to play functional roles in transnational city-to-city partnership establishments especially those between developed and developing countries [6, 38, 39]. Therefore, besides local governance, whether the civil society building, public participation encouragement, transaction cost decrease, and administrative efficiency improvement are also promoted by transnational city-to-city partnership? If so, what is the mechanism behind these promotions? Is the promotion mechanism dynamic in different stages and distinctive in Global East countries? Second, inspired by case studies on city-to-city partnerships between certain pairs of countries, the establishment of transnational partnerships is also a dynamic and unstable process. The partnership between cities in the US and South Africa is a steadily intimate process, while the partnership between cities in the US and Mexico sharply alienated due to the emergence of cross-border criminology [6, 8, 40]. Thus, what is the staged process of city-to-city partnerships between two specific countries? Does the staged process is sensitive to different pairs of cities? What are the factors that trigger, stimulate, obstruct, and even destroy the transnational partnership? These inquiries are calling for persistent scholarly endeavours.

## Supporting information

S1 Dataset City-to-city partnerships of 28 Chinese world cities from 1973 to 2019. (ZIP)

S2 Dataset List of city-to-city partnerships at different stages. (ZIP)

S3 Dataset City-to-city partnerships between 28 Chinese world cities and states at different stages. (ZIP)
